# Correlating Tumor Stiffness with Immunohistochemical Subtypes of Breast Cancers: Prognostic Value of Comb-Push Ultrasound Shear Elastography for Differentiating Luminal Subtypes

**DOI:** 10.1371/journal.pone.0165003

**Published:** 2016-10-24

**Authors:** Max Denis, Adriana Gregory, Mahdi Bayat, Robert T. Fazzio, Dana H. Whaley, Karthik Ghosh, Sejal Shah, Mostafa Fatemi, Azra Alizad

**Affiliations:** 1 Department of Radiology, Mayo Clinic, Rochester, MN, 55905, United States of America; 2 Department of Physiology and Biomedical Engineering, Mayo Clinic, Rochester, MN, 55905, United States of America; 3 Department of Internal Medicine, Mayo Clinic College of Medicine, Rochester, MN, 55905, United States of America; 4 Department of Laboratory Medicine and Pathology, Mayo Clinic, Rochester, MN, 55905, United States of America; University of South Alabama Mitchell Cancer Institute, UNITED STATES

## Abstract

**Purpose:**

The purpose of our study is to correlate quantitatively measured tumor stiffness with immunohistochemical (IHC) subtypes of breast cancer. Additionally, the influence of prognostic histologic features (cancer grade, size, lymph node status, and histological type and grade) to the tumor elasticity and IHC profile relationship will be investigated.

**Methods:**

Under an institutional review board (IRB) approved protocol, B-mode ultrasound (US) and comb-push ultrasound shear elastography (CUSE) were performed on 157 female patients with suspicious breast lesions. Out of 157 patients 83 breast cancer patients confirmed by pathology were included in this study. The association between CUSE mean stiffness values and the aforementioned prognostic features of the breast cancer tumors were investigated.

**Results:**

Our results demonstrate that the most statistically significant difference (*p* = 0.0074) with mean elasticity is tumor size. When considering large tumors (size ≥ 8mm), thus minimizing the statistical significance of tumor size, a significant difference (*p< 0*.*05*) with mean elasticity is obtained between luminal A of histological grade I and luminal B (Ki-67 > 20%) subtypes.

**Conclusion:**

Tumor size is an independent factor influencing mean elasticity. The Ki-67 proliferation index and histological grade were dependent factors influencing mean elasticity for the differentiation between luminal subtypes. Future studies on a larger group of patients may broaden the clinical significance of these findings.

## Introduction

Breast cancer is heterogeneous nature with several subtypes leads to differences in clinical treatment, outcomes and prognosis [1,2]. To establish an effective treatment and to predict the clinical course and outcome of breast cancer, it is important to use the most reliable prognostic factors. These prognostic factors include immunohistochemical (IHC) profiles (estrogen receptor (ER) status, progesterone receptor (PR) status, human epidermal growth factor receptor 2 (HER2) status and Ki-67 proliferation index) [3,4,5,6], lymph node involvement and histological grade [7] and tumor size [8]. Expression of (ER) and (PR) determines the responsiveness of tumors to hormone therapy and is currently used to select patients for such treatments [9,10,11,12]. The over-expression of HER2 status indicates a poorer prognosis [13,14]. Despite the numerous studies demonstrating its presence in proliferating cells, the exact role of Ki-67 in cell division is not well-known [5]. However, a higher Ki-67 index has been found to correlate with poorer prognosis and early recurrence. On the other hand, a lower Ki-67 index has been correlated with a favorable prognosis and late recurrence. Thus, Ki-67 proliferation activity may reflect the aggressive behavior of breast cancer; predict the time of recurrence, and the appropriate therapy required in treatment. Therefore, Ki-67 proliferation index must be considered in the treatment and follow-up of breast cancer patients [15].

Typically, a core biopsy sample is obtained to assess the histological and IHC features of breast cancer. It is known that changes in mechanical properties of breast tissue is correlated with disease progression, thus effecting treatment response and cancer risk [16]. Of these changes, accumulative abnormal deposition of extracellular matrix (ECM) in cancerous breast tissue progressively stiffens the stroma and has an important role in regulating the aggressive biology of breast cancer [16,17,18,19,20]. Thus, quantitative estimation of tumor stiffness can potentially add useful information similar to the prognostic features of heterogeneous groups of breast cancers.

Shear wave based tissue elasticity imaging is an ultrasound modality that provides quantitative measurements of tissue stiffness based on shear wave speed estimation [21,22,23,24]. Generally, malignant breast cancer tumors are stiffer than benign breast tumors and normal breast tissue [25,26]. Several published studies have reported that the aforementioned ultrasound modality can improve the accuracy of ultrasound, thereby helping to differentiate between benign and malignant breast tumors [22,27,28,29]. Denis et al. [22] utilized the comb-push ultrasound shear elastography (CUSE) to measure the elasticity of patients with suspicious breast masses. CUSE is a fast ultrasound-based quantitative and two-dimensional shear wave elasticity imaging technique. Recent studies have investigated the correlation between the shear wave measured tumor stiffness and IHC subtypes of breast cancer [30,31,32]. Chang et al. [30] reported the highest mean elasticity values in triple negative (TN: ER-,PR-,HER2-) tumors, which were significantly higher than ER+ subtype tumors. On the other hand, HER2 positive tumors showed higher mean elasticity values than ER+, but lower values than TN tumors. Youk et al. [32] found significant difference with mean elasticity from the following breast cancer prognostic factors: tumor size (*p* = 0.013), histologic grade (*p* < 0.0001), and lymph node involvement (*p* = 0.018). Additionally, for the IHC profiles and subtypes Ki-67 (*p* = 0.009), and the TN (*p* = 0.009) showed significant difference with the mean elasticity value. However, no IHC profile or subtype of the cancers was independently correlated with mean elasticity. Similarly, Ganau et al. [31] found no significant difference between the IHC profiles or subtypes with maximum or mean stiffness values of the breast cancers.

Herein, the prognostic histologic features (cancer grade, size, lymph node status, and histologic type and grade) influencing the association between mean stiffness and IHC profiles of breast cancer are investigated. Of particular interest is the association between mean stiffness and luminal subtypes.

## Materials and Methods

### Patients

This study was performed from January 2013 to December 2015 under an approved protocol by Mayo Clinic Institutional Review Board (IRB). 157 female patients, 18 years and older, with suspicious breast lesion were undergone B-mode ultrasound and CUSE before biopsy. Out of 157 patients, a total of 83 biopsy proven breast cancer patients formed the cohort of this study. The other 74 patients were determined to have benign breast masses from biopsy results. A written signed informed consent with permission for publication, approved by Mayo Clinic IRB was obtained from enrolled patients. All of our patients had received a clinical ultrasound and mammography prior to participating in the study. In total 83 patients participated in this study. Some of these patients composed of our malignant cohort in previous publications [22,29], evaluating the clinical utility of CUSE to differentiate between benign and malignant breast masses as well as correlations with tumor histology.

### Working principle of CUSE

In the CUSE technique, shear waves are produced by multiple laterally-spaced acoustic radiation force (ARF) beams [33]. The center frequencies of the ARF beams are set to 4.09 MHz with pulse duration of 600 ms. The generated shear waves in the tissue are tracked by a compounding plane wave imaging method at a 5 MHz center frequency. Some of the generated waves interfere with each other constructively and destructively. A directional filter is used to extract the left-to-right (LR) and the right-to-left (RL) propagating shear waves from the interfering waves at each pixel [34]. The shear wave speed is calculated by a 1-D autocorrelation method [35] and time-of-flight algorithm [36]. The final shear wave speed map is obtained by averaging LR and RL speed maps. Additional details can be found elsewhere [22,23,29,33,34].

**Fig 1:**
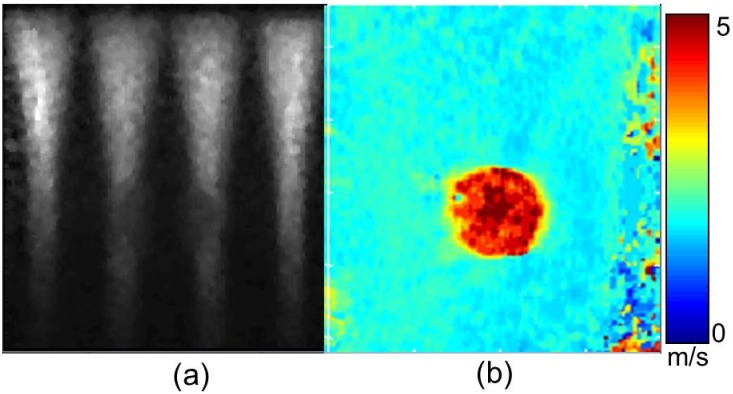
CUSE phantom study: (a) Acoustic radiation force excitation and (b) LR shear wave speed map.

In Fig 1 demonstrates the utility of CUSE on a CIRS spherical inclusion phantom (Model 059, Computerized Imaging Reference Systems Inc., Norfolk, VA, USA) was used for our phantom experiment. Conventional B-mode ultrasound and CUSE images were obtained using the Verasonics V1 system, (Verasonics Inc, Kirkland, WA, USA) equipped with a linear array transducer L7-4 (Philips Healthcare, Andover, MA). Fig 1(a) shows the CUSE excitation of the phantom using ARF beams, generating shear waves. The phantom has a background sound speed of 1540 m/s, ultrasound attenuation of 0.5 dB/cm/MHz, and density of 1030 kg/m^3^ with an inclusion shear wave speed of 1.73 times greater than the background. Fig 1(b) shows the LR reconstructed shear wave speed map. The color bar indicates the range of speeds on the shear wave speed maps.

### Conventional ultrasound and CUSE imaging

An expert sonographer with 29 years of experience in breast ultrasound conducted US imaging. Breast masses were localized using the B-mode US scanning, and then CUSE data were acquired prior to biopsy. CUSE produces a 2D full field of view (FOV) elasticity map with only one push-detect acquisition [33,34]. Additional information about performance of in-vivo CUSE for differentiation of breast and thyroid lesions are detailed elsewhere [22,23,33,34]. To calculate the stiffness values for a specific location, a region of interest (ROI) was drawn by freehand on the B-mode image. The ROI was automatically replicated on the overlaid speed map. Mean and standard deviation of the shear wave speed within the ROI were obtained. The measured shear wave speed was translated into elasticity (Young’s Modulus) in terms of kilopascals from the expression

$$E=3\rho c_{s}^{2}$$ (1)

where *ρ* = 1000kg/m^3^ represents the tissue density and *c*_*s*_ is the shear wave speed. In each case, the pathology of the masses was determined by clinical biopsy.

### Data analysis

The offline data analysis was performed using a graphical user interface developed with Matlab (MathWorks Inc., MA, USA). The mean shear wave speed was obtained within the selected ROI on the speed map after image processing. The processing includes using the normalized cross-correlation coefficient of the shear wave speed map as a quality control factor to reject pixels with unreliable speed measurements, as well as applying a mean filter to smooth the shear wave speed map. The shear wave speed map is displayed by a color map indicating shear wave speeds ranging from 0 to 8m/s. Tissue stiffness estimates are obtained within the ROI from the overlaid shear wave speed map. The elasticity value of a breast mass is calculated as the Young’s modulus of the mean shear wave speed within the ROI.

### Histological evaluation

The malignant pathology of the breast masses were confirmed by biopsy. The histological grade was determined using the method of Elston and Ellis [7]. The biomarkers ER, PR, and HER2 expressions were evaluated by the avidin-biotin complex staining methods. HER2 expression was initially assessed by staining, and tumors with equivocal HER2 results were further evaluated by fluorescence in situ hybridization (FISH). The St. Gallen International Expert Consensus 2011 proposed a new classification system for the breast cancer IHC subtypes [4]. According to these criteria, IHC markers including luminal A (ER+, PR+, HER2-, and Ki-67 low), Luminal B (ER+, HER- and either Ki-67 high or PR-), luminal B-like (ER+, HER2+, any Ki-67, and any PR), HER2+ (ER-, PR-, and HER2+), and triple negative (TN) (ER-, PR-, and HER2-) are used to differentiate invasive breast cancer subtypes [37,38,39,40]. Although, luminal A and luminal B are both ER+ and HER2- tumors they both display contrasting behavior [13]. Luminal A is defined by the cutoff value Ki-67 proliferation index < 14% as recommended by the 13^th^ St. Gallen International Breast Cancer Conference [41]. Luminal B subtypes show higher Ki-67 proliferation index, lymph node positive status, patient relapse, BRCA 2 mutations [42], and overall have a poorer prognosis [43].

### Statistical analysis

The relationships between quantitative values of mean stiffness, histological and IHC features were compared. In addition, clinical histological and radiological variables were compared among the tumor subtypes. Statistical analysis was performed using Matlab (MathWorks Inc., MA, USA) software. The Mann–Whitney U test was used to assess the association between mean tissue stiffness and histological characteristics of the malignant tumors. Two-tailed *p* values of less than 0.05 were considered to indicate statistical significance. The receiver operating characteristic (ROC) analysis was performed to determine the optimal cut-off value for differentiating luminal subtypes.

## Results

### Tumor size, histological features and immunohistochemical profile

The mean size of the 83 breast masses were 19.5 mm (range 4–75 mm), with an average of 108 ± 41.7 kPa mean stiffness value. In Table 1, when cancers were grouped by sizes of < 8 mm, 8–16 mm and > 16 mm larger tumors (≥ 8 mm) had higher mean stiffness values. A significant difference (*p* = 0.007) was found between small (< 8 mm) and large (≥ 8 mm) tumors with mean elasticity values. There was no significant difference found between 8–16 mm and > 16 mm tumor size.

**Table 1:** Correlation of shear wave elastography results with pathologic characteristics. Abbreviations: DCIS = ductal carcinoma *in situ*; IDC = invasive ductal carcinoma; ILC = invasive lobular carcinoma; IDLC = invasive ductal-lobular carcinoma; ER = estrogen receptor; PR = progesterone receptor; HER2 = human epidermal growth factor receptor 2; TN = triple negative.

		Number of patients	Mean stiffness (kPa)	*p* value
**Tumor size (mm)**				
< 8		5	58.7 ± 36.4	0.0074
8–16		43	104.4 ± 40.5	
> 16		35	121.1 ± 38.4	
**Histological grade**				
Grade I		19	100.3 ± 39.5	
Grade II		34	111.7 ± 40.4	0.493
Grade III		25	112 ± 42.1	
**Histological type**				
DCIS		3		0.741
IDC		50	108.5 ± 42.9	
ILC		8	119.7 ± 32.5	
IDLC		20	109.1 ± 39.7	
**Lymph node status**				
Positive		20	112.8 ± 39.9	0.912
Negative		57	110.9 ± 39.2	
**ER status**				
ER+		71	111 ± 39.3	0.723
ER-		11	101.1 ± 52.3	
**PR status**				
PR+		72	113 ± 38.9	0.15
PR-		10	86.7 ± 56.5	
**HER2 status**				
HER2+		15	120.4 ± 28.2	0.174
HER2-		63	106.1 ± 43.2	
**Ki-67**				
All tumor sizes	≤20%	44	99.9 ± 43	0.019
	>20%	30	122 ± 37.8	
	≤14%	38	100 ± 43.04	0.529
	>14%	36	117 ± 37.84	
≥ 8mm	≤20%	39	105 ± 41.29	0.0752
	>20%	30	122 ± 37.84	
	≤14%	34	104 ± 43.54	0.0976
	>14%	35	120 ± 36.06	
**Tumor size** ≥ **8 mm**				
Luminal A		30	108 ± 39	
Luminal B		17	125.2 ± 29.2	
Luminal B-like		11	123.8 ± 32.2	0.171
HER2+		3	115.4 ± 5.18	
TN		3	44.6 ± 61.8	
Unclassified		14	109.5 ± 47.5	

After biopsy, tumors were classified as ductal carcinoma in situ (*n* = 3) and invasive carcinoma (*n* = 78). The mean stiffness was 111 ± 62.7kPa and 109.8 ± 40.8kPa in invasive ductal carcinomas (DCIS) and invasive carcinomas, respectively, showing no significant correlation. There was one case of mucosa-associated lymphoid tissue lymphoma (25.2kPa) and one case of mucinous carcinoma (98.8kPa). Although, the invasive lobular carcinomas (ILC) had the highest mean stiffness (Table 1), there was no significant difference among the cancer histological types with mean elasticity values.

**Fig 2:**
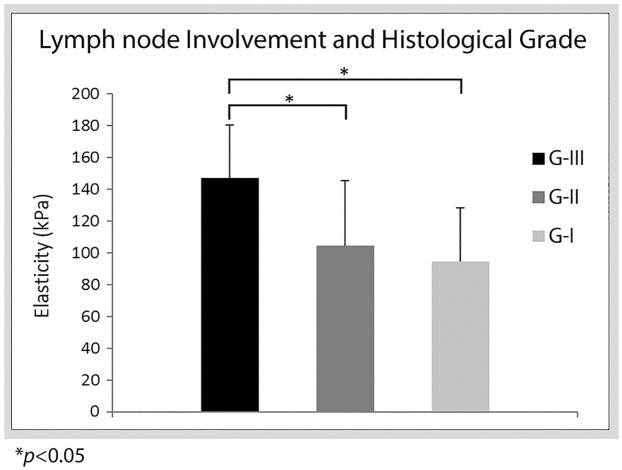
Bar plots of mean stiffness for histological grades I, II and III with positive lymph node involvement.

No significant difference was found amongst histological grades with mean elasticity values. Tumors with grade I (100.3 ± 39.5kPa) had a lower mean stiffness in comparison to grade II (111.7 ± 40.4kPa) and grade III (112.2 ± 42.06kPa) tumors, as shown in Table 1. However, for tumors ± 8 mm the mean stiffness for tumors with positive lymph node involvement was higher for histological grade III (*n* = 5 and 147.2 ± 33.7kPa) in comparison to grade I (*n* = 5 and 94.7 ± 24.7 kPa) and grade II (*n* = 10 and 104.6 ± 41kPa) is shown in Fig 2.

In correlation to ICH biomarkers, mean elasticity values of cancer masses showed no significant differences to ER, PR and HER2 status. As shown in Table 1, ER+ (111 ± 39.3kPa) and ER- (101.1 ± 52.3kPa) status have a *p* = 0.72 difference in mean elasticity values. Tumors of PR+ (113 ± 38.9kPa) and PR- (86.7 ± 56.5kPa) status have a *p* = 0.15 difference in mean elasticity values. Tumors of HER2- (106.1 ± 43.2kPa) and HER2+ (120.4 ± 28.2kPa) status have a *p* = 0.17 difference in mean elasticity values. Tumors with proliferation marker Ki-67≤14% and Ki-67>14% had no significant difference with mean elasticity values (*p* = 0.529). On the other hand, tumors with proliferation marker Ki-67≤20% (99.9 ± 43kPa) and Ki-67>20% (122 ± 37.8kPa) have a *p* = 0.019 difference in mean elasticity values. However, when only considering tumors size ≥ 8mm (*n* = 79) Ki-67 proliferation index has no significant difference with mean elasticity (*p* = 0.075 and *p* = 0.027, respectively), as shown in Table 1.

### Immunohistochemical subtypes

Since large tumors (size ≥ 8mm) showed no significant association with mean elasticity values, they were selected for our IHC subtype analysis. The large tumors were divided into luminal A, luminal B, luminal B-like, HER2+ and TN subtypes. The mean stiffness of the IHC subtypes is summarized in Table 1. The luminal B subtypes had the highest mean stiffness (125.2 ± 29.2kPa). There was no significant difference found between luminal A and luminal B subtypes with mean elasticity values. Similarly, no significant difference was found between luminal A and luminal B-like subtypes. Since there were only a few cases of TN and HER2+ tumors, they were not included in the analysis.

The relationship between the luminal subtype tumors and histological features with mean stiffness are summarized in Table 2. The grade I tumors were mainly composed of luminal A (*n* = 17) subtypes in comparison to luminal B-like (*n* = 2), no grade I tumors were composed of luminal B subtype. The composition of the grade II tumors were *n* = 11 luminal A, *n* = 10 luminal B, and *n* = 3 luminal B-like. The grade III tumors were luminal A (*n* = 1), luminal B (*n* = 7) and luminal B-like (*n* = 6) subtypes. Luminal B grade III tumors showed higher mean stiffness (128.5 ± 24.3kPa) than luminal A grade I (96.9 ± 40.4kPa) tumors. There was a significant difference (*p* = 0.039) between the grade I luminal A and grade III luminal B subtypes. The luminal subtypes were categorized as IDC (*n* = 35), ILC (*n* = 7) and IDLC (*n* = 15) histological types. There were *n* = 7 luminal subtypes with other histological types and *n* = 14 unclassified cases. The composition of the IDC luminal subtypes were *n* = 14 luminal A, *n* = 11 luminal B, and *n* = 10 luminal B-like. The IDLC luminal subtypes were mainly luminal A (*n* = 12) compared to luminal B (*n* = 3). There were *n* = 3 cases for each luminal A and B subtypes with ILC histology and only one luminal B-like subtype. No significant difference in mean elasticity value was found when comparing the histological types. In terms of lymph node involvement, there were *n* = 8 luminal A, *n* = 6 luminal B, and *n* = 3 luminal B-like tumors. The mean stiffness for the luminal B (115.8 ± 32.3kPa) and luminal B-like (152.1 ± 10kPa) tumors were higher in comparison to the luminal A (100.4 ± 25.1kPa) tumors. Of the luminal B subtypes positive for lymph node involvement, 66% (n = 4) have a Ki-67 proliferation index > 20% with high mean stiffness (125.1 ± 37.5kPa).

**Table 2:** Mean stiffness values of histological features compared to immunohistochemical subtypes (≥ 8mm). The number in parenthesis “( )” denotes the number of patients.

	Luminal A (kPa)	Luminal B (kPa)	Luminal B-like (kPa)
**Histological grade**			
Grade I	96.9 ± 40.4 (17)	-	129.2 ± 6.3 (2)
Grade II	120.8 ± 33.9 (11)	122.9 ± 30.6 (10)	127.8 ± 24 (3)
Grade III	96.5 (1)	128.5 ± 24.3 (7)	120.4 ± 37.4 (6)
**Histological type**			
IDC	102.3 ± 34.5 (14)	131.4 ± 25.1 (11)	126.7 ± 30.7 (10)
ILC	139.2 ± 17.7 (3)	119.6 ± 34.7 (3)	94.42 (1)
IDLC	106.5 ± 44.5 (12)	108.4 ± 24 (3)	-
**Lymph node involvement**			
Positive	100.4 ± 25.1 (8)	115.8 ± 32.3 (6)	152.1 ± 10 (3)
Negative	111.4 ± 42.8 (21)	130.3 ± 24.4 (11)	113.2 ± 29.1 (8)

**Fig 3:**
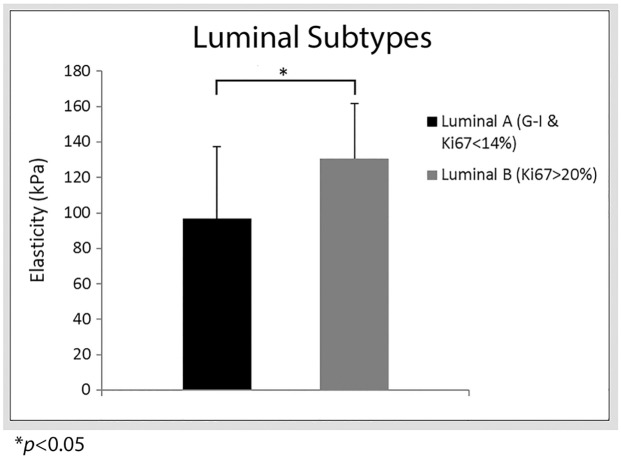
Bar plots of mean stiffness values for luminal A and luminal B subtypes. Young’s modulus values are reported on the y-axis and luminal type on the x-axis. Error bars show positive standard deviation.

A boxplot comparison of the large tumors for luminal A of grade I (*n* = 17) and luminal B Ki-67 proliferation index > 20% (*n* = 11) subtypes is shown in Fig 3. It shows that luminal B Ki-67> 20% has a higher median stiffness value than luminal A (grade I) subtypes. An ROC analysis between luminal A grade I and luminal B for Ki-67 > 20% yields the optimal cut-off value of 108 kPa with 72% sensitivity, 70% specificity and 73% area under the curve. There was a statistical significance of *p* = 0.036 between luminal A (grade I) and luminal B Ki-67> 20% subtypes. Similarly, a slightly higher significant difference (*p* = 0.039) was obtained between luminal A (grade I) and luminal B (grade III) subtypes with mean elasticity values. No statistical significance was found between luminal A (grade I) and luminal B-like subtypes.

### Review of selected cases

**Fig 4:**
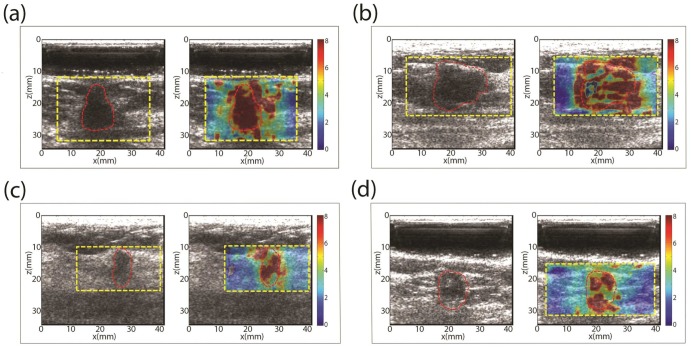
B-mode ultrasound (US) and shear wave speed map of of selected patient studies.

The results of four patients are individually reviewed:

**Case 1.** In Fig 4a shows a 12 mm mass in the greatest dimension and grade III invasive ductal carcinoma. The mean and standard deviation of the shear wave speed within the ROI was measured as 7.60 ± 0.84 m/s, which yields a Young’s modulus of 150.8 kPa. The immunohistochemical results show that the mass is ER+, PR+, HER2- and Ki-67 = 77.2%; IHC luminal B subtype.
**Case 2.** In Fig 4b shows a 14 mm mass in the greatest dimension and grade II invasive ductal carcinoma. The mean and standard deviation of the shear wave speed within the ROI was measured as 6.30 ± 1.67 m/s, which yields a Young’s modulus of 119 kPa. The immunohistochemical results show that the mass is ER-, PR-, HER2 ±; IHC TN and HER2+ subtype.
**Case 3.** In Fig 4c shows a 20 mm mass in the greatest dimension and grade III invasive ductal carcinoma. The mean and standard deviation of the shear wave speed within the ROI was measured as 6.34 ± 1.89 m/s, which yields a Young’s modulus of 120 kPa. The immunohistochemical results show that the mass is ER+, PR+ and HER2+; IHC luminal B-like subtype.
**Case 4.** In Fig 4d shows a 16 mm mass in the greatest dimension and grade II invasive ductal carcinoma. The mean and standard deviation of the shear wave speed within the ROI was measured as 6.16 ± 1.64 m/s, which yields a Young’s modulus of 113.8 kPa. The immunohistochemical results show that the mass is ER+, PR+ and HER2-; IHC luminal A subtype.

## Discussion

Our results demonstrate that mean elasticity showed the most statistically significant difference (*p* < 0.01) with tumor size. Larger tumors had a higher mean elasticity while smaller tumors were softer. This is concordant with previous studies [1,30,31,44,45,46]. Choi et al.[1] found a significant different (*p* = 0.017) in maximum elasticity with tumor size. Evans et al. [44] showed a *p* < 0.0001 significant difference in elasticity with tumor size, and found that larger tumors have higher mean stiffness values. Significant relation was found between mean stiffness and histologic grade amongst all tumor sizes and large tumors. When only considering lymph node positive large tumors, our results demonstrate grade III tumor had higher elasticity than grade I and II tumors. In previous studies, significant association of high histological grade with stiffness has been observed [30,32,44]. However, a recent study by Ganau et al. [31], found no significant difference in tumor stiffness with histological grade. Further investigation is needed to resolve this discrepancy.

Recent studies have investigated the association between the IHC profiles of the tumors and mean stiffness. Youk [32] and Ganau [31] found no significant difference in mean stiffness values among IHC profiles. Chang et al.[30] reported higher stiffness values for HER2+ and TN than ER+ (luminal subtypes). In addition, the study found significance in ER status and PR status biomarkers with mean stiffness. In our results, no significant difference was found among the biomarkers ER, PR and HER2 status. However, a significant difference (*p* < 0.05) was found between luminal A grade I and luminal B subtypes with Ki-67 > 20% as well as those a high grade with mean stiffness value. This is corroborated by atomic force microscopy measurements which demonstrated that ECM stiffness was significantly higher in luminal B samples than luminal A samples [20]. Atomic force microscopy (AFM) studies, the most well-known microscale-to-nanoscale tissue elasticity imaging technology able to monitor changes in ECM elasticity, demonstrated that the distribution of stromal stiffening increased from luminal A to luminal B subtypes [20,47]. Although we had a limited number of luminal HER2- samples, our observations that luminal B has higher stiffness than luminal A is in good agreement with other elastography [31,32] and AFM studies [20,47]. Our significant value for differentiating between luminal subtypes (*p*< 0.39) is concordant with the significant values of direct ECM stiffness measurements using AFM (*p*< 0.05) to differentiate between luminal A and luminal B for 25 samples per subtype [20,47].

Tumor cell cytokines, such as transforming growth factor beta (TGF-beta), have been used as surrogate markers for ECM stiffness. Increased levels of TGF beta stimulate cell migration and induce ECM deposition, remodelling and cross-linking to stiffen the extracellular stroma [48,49]. Although AFM measured ECM stiffness was higher for luminal B than A. Acerbi et al. [47] conducted an AFM study of breast tumors demonstrating positive correlation (*R*^*2*^ = 0.71) between increasing stroma stiffness and the level of cellular TGF beta signaling, but pSMAD (a marker of TGF-beta signaling in the cell [50]) showed an inverse relationship to ECM stiffness when differentiating between luminal A and luminal B subtypes. The level of pSMAD was higher in luminal A than luminal B which was the inverse relationship observed in ECM stiffness results. Since luminal differentiation is of particular focus of our manuscript, TGF-beta was not included in our analysis.

Although the numbers of patients with luminal subtypes were adequate for statistical analysis, the small number of HER2+ and TN tumors due to low prevalence is a limitation in our study. Also, the number of luminal B-like tumors was too small for a definitive conclusion. Similarly, the relationship between IHC subtypes and tissue stiffness for small tumors (< 8 mm) requires further investigation.

## Conclusions

Patient biopsies and CUSE interrogation of breast cancers suggest that tissue stiffness measured on the macro-level can distinguish luminal A and luminal B tumors when considering the subtypes Ki-67 proliferation index and histological grade. Therefore, tumor stiffness may parallel future disease progression along with size, grade and subtype as independent factors influencing tumor mechanics and predicting clinical outcome in patients.
